# “Social malaise... demonic agitation:” anarchism according to the criminology of the French physician Alexandre Lacassagne

**DOI:** 10.1590/S0104-59702023000100002en

**Published:** 2023-04-03

**Authors:** Bruno Corrêa de Sá e Benevides

**Affiliations:** i Doctoral candidate, Graduate Program in the History of Science and Health /Casa de Oswaldo Cruz, Fiocruz. Rio de Janeiro – RJ – Brazil brunoebenevides@gmail.com

**Keywords:** psychiatry, criminology, anarchism, political offence

## Abstract

This article analyzes the way anarchism and its followers were understood in *L’assassinat du président Carnot*, by the French physician Alexandre Lacassagne. A few months before the book was published, in June 1894, the president of France, Sadi Carnot, had been killed by the Italian anarchist Sante Geronimo Caserio. Lacassagne was called upon to perform the autopsy of Carnot’s body and a psychiatric examination of Caserio. The results of these two analyses were published in the aforementioned book. He made his observations on the anarchist in the broader context of criminological debates pursued in the late nineteenth century, which were not restricted solely to the authors of Italian criminology.

On June 23, 1894, a baker of Italian origin set off on a journey to Lyon from Sète, a small town in southern France, with the aim of avenging the death of comrades of his executed by the French judiciary for their involvement in a series of bombings of concern to the authorities.^1^ The target he chose was none other than the fifth president of the Third Republic, Marie François Sadi Carnot (1837-1894) (Assassinat..., 25 jun. 1894).

Sante Geronimo Caserio (1873-1894), as the baker was called, was just 20 years old. Of humble origins, he had become an anarchist militant after coming into contact with libertarian ideas. He arrived by train at the Vienne commune on the morning of the following day, having ensconced in his clothing a dagger with an ornate wooden handle and a Damascus steel blade some 28cm long by 25mm wide, which he had acquired with his own savings a few days earlier (Lacassagne, 1894, p.18).

From Vienne, a small commune 30km from Lyon, Caserio continued on foot to Lyon. At nightfall, he made his way to rue de la République, a street in the center of the city between the Saône and Rhône rivers. There, he took his place among the excited crowd assembled there to see President Sadi Carnot, who was on a routine visit to the region, as he passed down the street with his entourage. When his vehicle finally came near, Caserio moved forward, stepped onto the step at the side of the carriage and with his left hand pulled out the dagger from within his clothing. With a swift downward movement, he plunged the blade into the victim’s chest, damaging his heart and his portal vein near the liver (Lacassagne, 1894, p.14-16).

With his hands covered in blood, Caserio then cried out “Long live the revolution!” and “Long live anarchy!” until he was pinned down by the president’s bodyguards, who arrested and detailed him. Still alive, President Carnot was rushed off and underwent minor surgery, but to no avail, dying a few hours after the attack. The news of the attack was soon reported in the world’s press. The French newspaper *Le Petit Journal* reported on the episode with wealth of detail (Assassinat..., 25 jun. 1894). *The New York Times* published the front-page headline “CARNOT KILLED ... Cesare Santo, an Italian Anarchist, the Murderer. The People of Lyons, Infuriated, Make An Attack on the Italian Consulate. An Italian restaurant sacked” (Carnot..., 25 jun. 1894, p.1).^2^ In Brazil, *O Paiz* emblazoned its front cover with a report on the assassination, claiming that the attack had been the “criminal” work of anarchists, “a horde of madmen” (Sadi..., 26 jun. 1894, p.1).

The autopsy of Carnot was performed by a select group of French physicians, which the famous criminologist and leading exponent of French criminology, Alexandre Lacassagne (1843-1924), was invited to join.^3^ In addition to the forensic analysis of the victim’s body, Lacassagne took the opportunity to issue an opinion on the mental state of Caserio and the activities associated with anarchism, a movement of a socialist bent that, in the late nineteenth century, was becoming increasingly radical and widespread among the workers of several countries, constituting a transnational, internationalist movement (Jacob, Kessler, 2021).

Lacassagne’s analyses were collected and published in the book *L’assassinat du président Carnot* (Lacassagne, 1894), whereupon it became a respected source for understanding his criminological propositions about anarchists, especially those willing to take the most radical actions. It is worth mentioning that this work is regularly cited in works addressing both the study of the history of French criminology in the 1800s and the intellectual and scientific trajectory of Alexandre Lacassagne himself (Artieres, 1994; Renneville, 2003, p.233, 2005). However, in such works his book is addressed only superficially, without examining Lacassagne’s ideas about militant anarchists in any detail.

Accordingly, this article analyzes this small work by Lacassagne, extracting his main observations about Caserio and more widely about anarchism. The aim is to show how this medical and criminal forensic study of the anarchist was part of a wider debate in the field of criminology and forensic psychiatry in the late nineteenth century, which sought to introduce libertarian militants into broader discussions about crime and madness.

From the theses Lacassagne put forward in *L’assassinat du Président Carnot*, it can be understood that there were a number of criminological theories that addressed anarchists and their actions in circulation at the time. This then enables a new perspective to be taken on historiographies from Brazil (Samis, 2002; Lopreato, 2003; Avelino, 2010; Monteiro, 2010) and elsewhere (Pick, 1989, p.109; Jensen, 2001, 2004; Ansolabehere, 2005; Sierra, 2009; Knepper, 2017; Salvatore, 2017) that have given precedence to the role of Italian criminal anthropology in the context of anarchy, especially the propositions of Cesare Lombroso (1835-1909) formulated in *Il delitto politico* (1890) and *Gli anarchici* (1894) (Calafato, 2013). Despite the importance of these publications, they end up overshadowing the work of French criminologists – especially Lacassagne – concerning anarchism and political felonies. This is the gap that this article proposes to discuss and fill.

To this end, this study situates the history of criminology in the field of intellectual history in connection with the history of science.^4^ As for its theoretical methodology, the “evidential paradigm” systematized by the Italian historian Carlo Ginzburg (1989) is adopted. Considering the textual nature of *L’assassinat du président Carnot*, its evidence was studied for homogeneity, meaning, and logic to enable a historicization and interpretation of Alexandre Lacassagne’s criminological concepts and ideas about anarchists.

The article is divided into three sections. The first discusses the broader circumstances of the anarchist movement in central Europe at the time when Caserio carried out the attack. The second presents an analysis of how criminology was organized in the 1890s, putting particular emphasis to the work of Lacassagne and other French criminologists. The third focuses on *L’assassinat du président Carnot*, in which Lacassagne writes about the attack on President Carnot and makes a profound investigation of the practice of anarchism.

## Daggers and dynamite: the radicalization of anarchism in continental Europe

The anarchist movement gained prominence as of the mid-1800s, once its main ideas had taken shape, and soon spread to different countries (Taibo, 2018). By the 1880s and 1890s, anarchist militancy had taken on very radical overtones. This was directly related to the strategy known as “propaganda by action,” which had been defended by some anarchists as early as the mid-1870s.

Propaganda by action was a strategy of insurrection advocated by the Italians Carlo Cafiero (1846-1892) and Errico Malatesta (1853-1932), the latter of whom was a leading exponent of anarchism, which was also supported by the Russian anarchist Piotrpot Krokin (1842-1921). This revolutionary strategy aimed to “teach socialism through facts, through the lessons of things” (Marini, 2017, p.336). The idea was therefore to spark a revolt, like an armed uprising or a general strike: something that would be strong enough to incite the workers and thus pave the way for social revolution.

However, in the 1880s and 1890s, propaganda by action ended up extrapolating its initial definition, and staging attacks against public figures and in places frequented by the upper bourgeoisie gradually became the central objective of the supporters of this strategy (Jacob, Kessler, 2021, p.9-10). Indeed, some of the anarchists even concluded that adopting this *modus operandi* would render the need to form large organizations obsolete, since violent acts of this nature could perfectly well be planned and executed by small clandestine groups. Still, some activists, such as Kropotkin and Malatesta, continued to defend the need for both types of organization, since larger ones had the power to rally workers and carry out libertarian propaganda (Jensen, 2004; Cahm, 1989, p.115).

In 1881, a congress was held in the City of London that was attended by delegates from various parts of Europe and America. Despite the participation of militants of different anarchic orientations, the meeting was marked by the superimposition of radical ideas that would influence some branches of anarchism for almost two decades (Farré, 2012, p.161, 166). In theory, the intention of some anarchists, such as Kropotkin, was to have small clandestine groups make use of violence in order to “attain revolutionary goals” (Sierra, 2003, p.196), while a larger organization would provide aid, resources, and other forms of support. In practice, what ended up happening was that a spate of attacks was set off by individuals working alone; that is, they declared themselves anarchists without having any kind of organic bond with the federations (Joll, 1977, p.148-149).

So it was that a series of attacks by more radical activists took place at the very same time that the leading lights of anarchism were debating theoretical questions about the use of violence. The targets of these attacks, carried out in the late 1870s and early 1880s, were public figures in several parts of Europe. According to Jensen (2004, p.125), most of these assassination attempts were not made directly by anarchists, but as they came to be defended in libertarian circles, they were quickly held accountable as the unequivocal perpetrators of whatever action of this nature may occur, including some episodes with no political motivation whatsoever.

The wave of attacks peaked in the 1890s, recurring in different locations and under the pretext of being a response to the economic, social, and political reality experienced by the proletariat in the main European capitals. For Daniel Colson (2017, p.181), the use of violence as propaganda also reveals the difficulty radical movements faced in obtaining social recognition for their ideologies and demands in the political sphere. For the authorities, the most emblematic acts at this time were the assassinations of Empress Elizabeth of Austria, in 1898, and of William McKinley, president of the United States, in 1901, both by individuals who claimed to be anarchists. The episodes caused great commotion in the world press and caused concern in many countries, prompting them to address the issue head-on (Jensen, 2004, p.117).

In France, where anarchist ideas had gained prominence among workers, this radical turn was marked by the publication of small newspapers and pamphlets by different anarchist groups to spread “incendiary” articles, songs, and poetry. An example of the extreme action includes a sequence of terrorist acts carried out under the influence of an attack by Ravachol in March 1892.^5^ From this year to June 1894, there were a total of 11 dynamite explosions in Paris, taking the life of nine people (Merriman, 2009; Woodcock, 2006, p.80).

In November 1893, an anarchist cobbler Léauthier seriously injured a Serbian minister on a diplomatic visit to Paris. One month later, Auguste Vaillant threw a bomb in Palais Bourbon, home of the lower house of the French legislature, in revenge for the execution of Ravachol. In February of 1894, Émile Henry lobbed explosive material into Café Terminus, also in the French capital. And the following month, the Belgian Amédeé Pauwels died when some dynamite intended for an attack on the Madeleine church went off accidentally (Maitron, 1981, p.12-14). In April 1894, shrapnel from a bomb thrown in the Foyot restaurant injured the French poet Laurent Tailhade in the eye. In June of the same year, it was the turn of the Italian Sante Geronimo Caserio, who, as described, traveled to France to avenge the death of some French and Spanish anarchists executed by their respective governments (p.12-14).

As this period of extreme anarchism assailed Europe, concern with these occurrences prompted physicians, jurists, and other intellectuals involved in researching criminals to publicize their work in order to sketch out a scientific explanation for the “attraction” anarchy exerted over some subjects. These studies took place in the context of the development of European criminology in the late nineteenth century.

## Medicine, criminology, and theories about crime

In addition to the proletarian uprisings raging in the main European capitals, another social phenomenon was a cause for concern among the political elites of the nineteenth century. The economic contradictions spawned by capitalism were not only awakening political resistance on the part of the working class, but were also responsible for a vertiginous increase in urban crime (Hobsbawm, 2012, p.318-322).

Even in the early decades of the nineteenth century, increased levels of recidivism within city limits had stimulated numerous reflections were about crime and criminals. These studies were produced by “social scientists” from different fields and put forward different approaches to managing crime using statistics and studies into the profile of the criminal classes (Horn, 2003, p.8). Although these analyses highlighted the role of individual factors in criminality (e.g., sex and age), none of them went so far as to characterize criminals as “biologically different from the general population” (Wetzell, 2000, p.28).

As of the mid-1800s, however, this topic also attracted the interest of physicians keen to understand “deviant” subjects and mental ailments, which resulted in the formulation of new theoretical explanations for crime. While they attributed multiple causes to criminal acts, the strongest focus of these theories was on the bodily and psychic abnormalities of “criminals.” As Marc Renneville (2003, p.204) points out, in the light of the medical gaze, crime changed status: “It was no longer conceived as a sin or a failure, but as an irrational act, a kind of ‘malady’ that plagued the ‘social fabric’.” These studies contributed to the development of criminology as a field of scientific knowledge engaged in developing international itineraries, theories, practices, and agendas, impacting the ideas of thinkers from various regions of the world (Becker, Wetzell, 2006), including in Brazil (Alvarez, 2002; Ferla, 2005; Dias, 2015).

The upsurge in crime recorded in the last few decades of the nineteenth century brought to light the limitations of “classic criminal law”^6^ in dealing with the issue of delinquency, since it did not offer European political elites an explanation for recidivism and was unable to propose measures of a greater scope to fight crime apart from the administration of harsher penalties. It was in this context that the first medical criminological discourse developed, whose main purposes were to relativize the role of volition (free will) in the committing of crimes, make the criminal the central object of their analyses, propose multicausal factors for criminogenic behavior, and, according to Luis Ferla (2005, p.16), recognize sentences as a form of treatment and not just punishment.

Following these guidelines, most of these formulations sought to identify a variation of the human genre in criminals. To do so, its creators intended to organize a science that was capable of describing the biological inequalities that exist among men (Alvarez, 2002, p.680). This resulted in the construction of a highly specialized approach based on the observation of the skull, skin, organs, and bone structure to reveal potentially innate “inferiority” (Becker, 2006, p.112-113).

The attempt to correlate the physical characteristics of the body with a “propensity for crime” was nothing new, insofar as other branches of mental medicine, such as phrenology and cranioscopy, had already explored the issue. Developed initially by Franz Joseph Gall (1758-1828) and subsequently by his student of German origin Johann Spurzheim (1776-1832), these two medical sciences made important contributions to the criminological theories produced by both criminal anthropology and the French criminologists (Lanteri-Laura, 1994, p.22-23).

Throughout the second half of the nineteenth century, the science of criminology was a movement with different strands and approaches (Kaluszynski, 2006, p.310). In several analyses produced by social historians, these divergences have been characterized as a pure duality between two opposing “schools:” the French criminologists, advocating ethnographic and sociological approaches to crime, versus representatives of Italian criminal anthropology, described as overly concerned with the deviant bodies of criminals. In the more “Whiggish” narratives, biodeterministic theories are overcome and defeated by the French sociologists, who are more subtle and mindful of the social setting (Horn, 2003, p.3).

The American historian David Horn points out that discussions of this duality between the “Italian” and “French” schools were first raised in the debates on criminology back in the late nineteenth century. Horn (2003, p.4) also mentions that this bifurcation ended up being reinforced in the historiography from the 1970s and 1980s, which produced very reductionist interpretations. However, this opposition has since been contested by foreign researchers, who have relativized the differences between the two “schools.”^7^ These studies have shown that the French and Italian criminologists of the second half of the nineteenth century did in fact attribute criminal behavior to multiple factors, refuting the idea that any single cause was ever held as determining such behavior (Wetzell, 2000; Becker, Wetzell, 2006; Mucchielli, 2006; Knepper, 2017).

The first studies developed in the field of criminal anthropology were published in Italy by the physician Cesare Lombroso and the jurists Enrico Ferri (1856-1929) and Raffaele Garofalo (1852-1934) (Villa, 2013). In 1876, Lombroso published his most important work, *L’uomo delinquente*, in which he defended the “theory of atavism.” Years later, in 1899, he produced a compendium for the general public entitled *Le crime, causes et remèdes*, compiling all his ideas in the field of criminology (Gibson, 2006, p.141-142).

At first, Cesare Lombroso understood crime from the perspective of the aforementioned phenomenon of atavism. In other words, an offense was a kind of characteristic behavior of “inferior” humans, which may occasionally reappear in evolved social groups. There was therefore a systematic relationship between the “criminal man” and “prehistoric man” (or “savage man”). The roots of this atavism could be attested by the morphology of some parts of the body of delinquents, which Lombroso claimed were very similar to the those found in some carnivorous plants, rodents, primates, and also fetuses of *Homo sapiens* (Spierenburg, 2016, p.384; Knepper, 2017, p.54). A criminal act, he went on, was a reflection of the madness caused by an “atavistic animality” from which subjects could not escape. This conclusion served as the basis for the theory of “innate delinquency,” an expression used in allusion to individuals predisposed to the “world of crime.” However, the idea of atavism was harshly criticized by his opponents and was quickly abandoned. In later publications, Lombroso put forward some very eclectic etiological explanations for crime, which included organic, climatic, geographical, and social factors (Rafter, Posick, Rocque, 2016, p.70; Musumeci, 2018, p.86).

Despite the criticisms levelled against them, Lombroso’s studies became so popular that between the 1870s and 1940s they were appropriated, debated, and challenged in various countries. In line with other actors, Lombroso contributed actively to the constitution of criminology in a transnational, interdisciplinary field (Henze, 2009; Villa, 2013, p.10).

French criminology also took shape as of the second half of the nineteenth century. Like criminal anthropology, its proponents came from a variety of backgrounds, ranging from physicians, jurists, lawyers, and magistrates to social theorists and public figures. This diversity lent the group a quite plural character, resulting in the formulation of different theories about crime and criminals (Nye, 1984, p.98).

Its main representatives were to be found in Lyon. Their studies were published in the journal *Archives de l’Anthropologie Criminelle*, which circulated between 1886 and 1914 (Kaluszynski, 2006, p.303). The journal’s editor was the physician Alexandre Lacassagne, and the jurists René Garraud (1849-1930) and Gabriel Tarde (1843-1904) were also closely involved in the initiative.^8^

According to Marc Renneville, most of the French criminologists from this period rejected Lombroso’s theses of atavism and “innate delinquency.” While they did recognize the existence of anatomical or physiological abnormalities in criminals, they did not regard them as being frequent enough for any inference to be drawn as to their primitive (“prehistoric”) nature. Under the strong influence of the theory of degeneration and neo-Lamarckian ideas, they set great store by environmental conditions (poverty, poor working conditions, climate, alcoholism, diseases etc.) as the origins of deviant behaviors (Renneville, 2005, p.191).

The leading figure of French criminology in this context was Alexandre Lacassagne. He argued that pernicious social conditions had the power to alter the organic constitution of the brain over several generations, resulting in changes in behavior and socially “maladjusted” conduct. As a product of the social milieu, individuals with such a constitution also contributed to the formation of a harmful environment, in that they helped to propagate degeneration via hereditary transmission. Lacassagne therefore always considered biological factors in his analyses; but, unlike Lombroso, “he saw physical and psychic anomalies of criminals as the consequences of an unfavorable [degenerate] social environment ... and as not etiological factors of crime” (Renneville, 2005, p.193).

Between the late nineteenth and early twentieth centuries, some conferences on criminal anthropology were held in Europe, attracting the top experts on the subject. Eight international events of this nature were planned between 1885 and 1914, each hosted by a different city: Rome (1885), Paris (1889), Brussels (1892), Geneva (1896), Amsterdam (1901), Turin (1906), Cologne (1911), and Budapest (1914), although this last event did not take place (Kaluszynski, 2006; Henze, 2009; Del Olmo, 2017).

The first congress, in Rome, represented the culmination of Lombroso’s career and Italian criminology. However, the second event, held in Paris, was marked by strong opposition to the idea of *Homo criminalis*. The criticisms came from the “Lyon criminologists,” represented by Lacassagne. Although the friction between the French and Italians came to a head, it is worth bearing in mind that there was some common ground in the ideas formulated by these two groups, as mentioned above. As Martine Kaluszynski (2006, p.306, 307) notes, these meetings were places for interchange, scientific controversy, communication, and the development of medical-criminal knowledge, as well as conflicts of interests and power, “where adversaries ... either clashed or allied themselves” around specific theoretical propositions.

## “Human beast, defective in its origins:” the anarchist militant, according to Alexandre Lacassagne

The emergence of criminological discourses and the anarchist attacks in the late 1800s led to an increase in the number of studies and publications devoted to understanding the social origins of anarchism and, in most cases, setting about proving that the activities and ideas of anarchists went against the “foundations of social order.” Physicians and other specialists keen to shed light on “deviant” subjects turned their attention to “political delinquents,” social agitators, and revolutionaries in general. Influenced by theories that correlated crime with madness, they looked for a scientific solution to the “problem” that was the anarchist movement, especially in its most radical actions (Renneville, 2003). It was in this context that in 1894 Lacassagne published his *L’assassinat du président Carnot*, a work whose subject matter was very much in vogue at the turn of the century.

In this short, 111-page book published in Lyon by A. Storck, which had numerous titles in the field of criminology, Lacassagne began by addressing the reasons that had apparently led Caserio, an anarchist militant, to attack Carnot. First, he presented his concept of crime. For Lacassagne, criminal conduct should be understood as any “act detrimental to the existence of a human community.” Despite being quite generic, this definition had the advantage of typifying any conduct considered prejudicial to society “at any time in history” as an offense (Lacassagne, 1894, p.3).

Basing himself on this definition, Lacassagne went on to argue that the acts of anarchist militants should therefore be considered “essentially criminal.” These individuals’ desire to “change the functioning of ordinary life, the relations of capital and labor” was Lacassagne’s (1894, p.3, 4) main argument for the criminalization of anarchy. Moreover, he interpreted the ideas of having a society based on collective ownership and the sharing of goods produced as “aphorisms” advocated by “young men, almost children;” that is, by naïve people whose youth made them blind to the real and practical needs of life.

Lacassagne continues his analysis by investigating the origins of the ideals defended by the anarchists. Diverging from the position of other specialists, libertarians, in general, could not be understood as a group of subjects who were exalted or mentally imbalanced. Rather, he argued, anarchism was a social phenomenon, a malaise caused by emerging inequalities affecting the proletariat in large urban centers:

But where do these tendencies, these ideas, come from? It is not, as we often repeat, the state of spirit of a few individuals, more or less overexcited or unbalanced.

No, it is the sign of a social malaise resulting from several causes that we can glimpse, but which are difficult to pin down and whose influence cannot be specified. It is like the demonic agitation, possession, witchcraft, which occupied the entire Middle Ages. We were then concerned about the fate of the soul during this life, especially after death, but we accepted social inequalities. Today, it is the body ... that must be satisfied: we have necessities and we wish to enjoy the relationships of modern life (Lacassagne, 1894, p.4).

The desire to satisfy the “necessities” of life, however, could not be unrestricted to the point of producing discourses and ideas that encouraged the expropriation of goods, demanding the end of private property, as the socialist workers’ movements desired. Also, according to Lacassagne, such actions deserved due social disapprobation for being “the manifestation of selfish instincts” of individuals who desired only “their own well-being, to the detriment of others,” to which end they nourished ambitions “driven by destructive instincts” (Lacassagne, 1894, p.4).

Lacassagne (1894, p.5) held that there was no scientific basis for advocating the complete elimination of the principle of authority and political institutions or an end to a society based on competition. Anarchy meant individuals fighting for their rights, “but individuals in revolt against society, in rebellion against their own species.” Further, the idea of isonomy, another important precept advocated by libertarians, had been widely disseminated and accepted by “weak and superficial minds, who [saw] equality only in appearances” and had no understanding of the “natural diversities of men” (p.6).

As we can see, the anarchist typified by Lacassagne was a person of “unruly pride, full of feelings of rage and envy, in a state of chronic anger.” Among young people, this rage was reinforced by an “abhorrence of all authority, and particularly militarism.” However, Lacassagne admitted that the “evils of civilization” existing in big cities had the power to curb the “gentleness, courtesy and benevolence present in men,” causing “pride, or the instinct of domination, to progress and grow” to the point that individuals “no longer feel obliged to follow rules.” The “unruly” pride in turn acted on the destructive instinct, which explained “the language of violence and anarchist actions” (Lacassagne, 1894, p.7).

In 1890, Cesare Lombroso and the lawyer Rodolfo Laschi (1850-1950) published the book *Il delitto politico e le rivoluzioni in rapporto al diritto, all’antropologia criminale ed alla scienza di governo*. In it, they comment that political crime, in its anthropological sense, should not be understood only as an attack on a particular political institution. For them, the true essence of such crime was to establish “violent opposition to political, religious, or social misoneism,”^9^ which existed in most nations. Human progress was slow and encountered “powerful obstacles caused by external and internal circumstances.” Both individuals and society, they argued, were conceived as entities of a conservative nature. Thus, abrupt and violent actions designed to effect sudden social change were antisocial “and therefore, legally, a crime.” They also presented an already existing distinction between revolutions and uprisings. While the former were gradual, fair, and natural and, as such, lawful, the latter should be understood as precipitated and artificial, and thus unlawful. Moreover, revolutionary processes occurred in the zone of normality, while small uprisings were situated among pathological phenomena (Lombroso, Laschi, 1890, p.31).

The perception that insurrections were sudden and abrupt, and thus incompatible with human nature, was also explored and revisited by Lacassagne (1894, p.9). For him, “anarchists, these men of rapid progress who seek instant solutions,” end up exhibiting retrograde principles by defending the destruction of society. However, he argued, “the human brain is one and its development is so slow that it is almost immutable, just like in animal species.” Although “certain essential, inescapable, and primordial instincts in the brain” could change, this organ “will not change any more than the limbs and body of man.”

At the end of the section that explores the main etiological factors of anarchism and their interface with political crimes, Lacassagne (1894, p.9) is categorical: “The revolutionary spirit is the result of overexcited human egotism associated with poor education.” Referring to Gabriel Tarde’s studies on criminal mobs, he comments that such individuals are led to commit unlawful acts by suggestion. When newspapers, small periodicals, and pamphlets published by the libertarian press disseminated incendiary writings, they contributed to “the suggestion of political crime,” resulting in the “explosive combination of dynamite and daggers,” as in the case of Sante Geronimo Caserio (p.8).

In the third part of his work, Lacassagne analyzes the social trajectory of the assassin, the pathological history of his ancestors, his anthropometric data, and whether his contact with libertarian ideas was the result of a “deranged mind.” In so doing, he intends to raise hypotheses that might explain the criminal act and, above all, enable a conclusion to be drawn as to whether Caserio was insane or acted under the influence of strong emotion, both of which would protect him against criminal liability.

First, he considers his place of birth, in Motta Visconti, a small commune in the Lombardy region, southwest of Milan. He expresses the view that by the second half of the nineteenth century, Italy had become a “land of evil,” “the classic land of bloody crimes, or simply ... of impromptu killings.” The country’s *impromptu omicidio* was, he claims, “a common export item” to neighboring countries, especially France (Lacassagne, 1894, p.20).

Another etiological factor he considered in his analysis was Caserio’s youth (20 years old). Referencing the then famous study of 1890 on regicide (*Les régicides dans l’histoire et dans le présent*) by the physician Emmanuel Régis (1855-1918), Alexandre Lacassagne (1894, p.21) pointed out that, with rare exceptions, “all the famous perpetrators of regicide were just 30 years old at the time of the attack,” making an impression on “the large number of young people who participated in the anarchist movement.” In the anthropometric information on Caserio, collected in Saint-Paul prison, no abnormalities or “elements of degeneration” were found, merely the physical traits common to regicides, exactly as reported by Régis in his research, including: a thin beard, a fixed gaze, and a slightly elongated skull (p.25).

As for the social trajectory and “mental state” of the attacker, Lacassagne points out that Caserio “barely had primary education,” being “fluent in reading and deficient in writing.” His visual memory was excellent: he was able to recall “small facts of things experienced.” Noting the testimony collected in court, especially the handwritten statements, Lacassagne writes that “we were convinced that his wit was sufficiently keen, but superficial. He understood things quickly, but was unable to reflect, compare, and judge,” and his knowledge of politics was “almost null” (Lacassagne, 1894, p.25-27). Caserio underwent “forced feeding of readings, of which he had retained only a few sentences” that sounded “good to his ear.” For their “eminently simplistic” essence, the “incendiary” anarchist ideas were “enough food for his mind” and because they were easy to understand “he avidly read the newspapers and pamphlets of this vulgar and hotheaded sect” (p.28).

Again chiming with Tarde’s studies, Lacassagne argues that news about the executions of other convicted anarchists was responsible for “germinating in his brain the ideas of hatred and revenge.” The discourse spread through the libertarian press was so pessimistic that it made its readers feel “tired of life” and begin to consider the possibility of committing suicide. However, because “anarchic individuals” were possessed of “immeasurable vanity,” they sacrificed everything for their cause, “putting their heads on the line and showing their comrades” how “strong and resolute” they were. Thus, concludes Lacassagne (1894, p.29; emphasis in the original), Caserio was not a regicide: “his crime was ‘indirect suicide’.”

The category of “indirect suicide” had been formulated by Emmanuel Régis to distinguish it from true regicide. In this kind of act, a person who is “fearful and hopeless with life” kills a politician or a monarch with the aim of being condemned and executed, and thus indirectly achieving “his own death, which was his only goal” (Régis, 1890, p.22). On the other hand, the acts performed by the true regicides should not be seen as sudden or heedless. On the contrary, they were logical, “conceived with full lucidity, premeditated, and prepared over a long time.” Despite this distinction, these perpetrators were “sick, unbalanced, weak-willed, slaves to their obsession and, driven by a blind and fatal force, are not free to resist” (p.20).

Alexandre Lacassagne notes that Sante Caserio had never been “restrained or guided by the influence of friendship or love,” indicating that the humble baker from Lombardy “was frigid, uncouth, and insensitive to platonic love.” Indeed, in his indifference he had not expressed any sense of remorse or regret for the assassination. His limited education, “his limited wit, incapacity to elaborate an observation or reflection, and his complete absence of feelings of affection” revealed him to be impulsive. Caserio was therefore similar to “these individuals with muscles of steel” who therefore had “no fear of obstacles, were insubordinate, excitable, with blood like dynamite,” making them as dangerous as “bombs in the hands of anarchist leaders” (Lacassagne, 1894, p.31).

After tracing Caserio’s criminological profile, Lacassagne made a number of conjectures about his sanity. However, he preceded this with the view that “criminals of the like of Caserio are not mad” or completely degenerate. He also ruled out the possibility of his having epilepsy because he did not display any of the telltale signs:

But what kind of madness could he have? ... Is he prey to one of the forms of epilepsy? He is the son of an epileptic; this heredity is established, and everyone knows it is oppressive.

Therefore, let us ascertain whether Caserio has the symptoms or stigma.

He does not have the characteristics of epileptogenic asymmetry described by Lasègue. He does, however, have a rounded, dimpled chin. His face has no other particularities except for this continuous, contracted, satanic laughter, which produced a painful impression on me. Was that laughter affected by the circumstances, or was it usual for him? A similar, continuous laugh is a kind of tic. We must beware of those who cannot laugh: they are wicked and deceitful.

Recall that his girth far exceeds his height (Tonnini, Civadolli and Amati), that he has overlapping fingers (Lombroso), that when standing he has a stance reminiscent of that of quadrumans, with his feet apart to give himself a wider base for support (Féré).

... We never noticed any unconsciousness ... there is no trace of hallucinations, visions, nightmares, crises ... In sum, from the information we have, we can say that Caserio was not an epileptic (Lacassagne, 1894, p.32-33).

Summing up, Lacassagne concludes that “Caserio is not a fool” but that he is “possessed of some features of degenerates.” This case was one of a “fanatical killer,” “a human beast, defective in its origins” who was “corrupted by the theories of the anarchist party, which made him an antisocial being.” For Caserio, the assassination of President Sadi Carnot was “a means of terrorism, the revenge of a group, the satisfaction of hatred and, at the same time, the consecration of a reputation” among his comrades. On the basis of this opinion, he concluded that Caserio should be held criminally responsible, having acted in full possession of his faculties at the time of the event. As he could be held to account for the crime committed against the French head of state, it was “right and proper that he receive the punishment” reserved for such an offense in French criminal law (Lacassagne, 1894, p.37).

The anarchist Sante Caserio was prosecuted, convicted, and sentenced to death by the court. His execution took place on August 16, 1894. According to the press, Caserio walked slowly to the scaffold, holding firm, showing no rancor or weakness. Livid, he was assisted by two men to approach the guillotine. In their almost cinematic description, the papers reported that as the blade slowly pressed into his neck, “a hoarse voice, strangled by fear” could be made out, saying “courage, comrades, long live anarchy!” (L’execution..., 17 ago. 1894).

## Final considerations

This article sought to show that the growth of radical actions, such as the assassination perpetrated by Sante Caserio and explored in Alexandre Lacassagne’s book *L’assassinat du président Carnot*, brought anarchism to the center of the criminological debates taking place in France, Italy, Germany, Spain, and other parts of Europe in the second half of the nineteenth century. Based on theories associating crime with madness, the ideas Lacassagne expressed in the book aimed to give multifactorial explanations for the phenomenon of anarchism (such as race, heredity, and social factors), while also suggesting ways to curb the spread of libertarian ideas. Although Caserio was not declared insane, much of the analysis made by Lacassagne demonstrates an intention to fit the anarchist into the realm of mental “deviations.”

By the beginning of the twentieth century, libertarian ideas and practices had parted ways, affecting the forms of organization and action of the working classes. According to Bert Altena, the fact that anarchism was a social movement allows us to understand its adaptability and mutability in response to new social demands. This would also explain its “staying power in the long run,” notwithstanding some periods of latency (Altena, 2016, p.16). In this sense, after the cycle of “acts of terror,” the propositions initially spawned in the 1870s survived in the form of an anarchist movement in symbiosis with experiments in syndicalism. The interlocution of these two fields forged the foundations of “revolutionary syndicalism,” in which workers’ associations were organized around national or regional federations, adopting the same nonpartisan and internationalist model advocated in the First International (Colombo, 2004, p.28-29; Hirsch, Van der Walt, 2010, p.li).

One consequence of the attacks was a strong backlash against the anarchist movement in an effort orchestrated jointly by some countries, which signed international agreements aimed at the exchange of police information and the passing of laws criminalizing and expelling individuals who maintained some link with anarchism (Bantman, 2016). However, the many deportations of militants had the unintended consequence of fueling the circulation of libertarian ideas, resulting in the creation of a transnational network of activists in far-flung parts of the European continent, since many of the returnees were responsible for spreading anarchism on foreign soil (Romani, Benevides, 2021).

As another consequence, the specter of the “dynamite-wielding anarchist,” exploited to exhaustion by an emerging sensationalist press hungry for crime-related news (Kalifa, 2019), was instrumental in nurturing in the collective imagination the idea of a “madman” with a bag tucked under his arm containing an explosive device, ready to “blow everything to smithereens.” The third consequence caused by the excesses of propaganda by action was an increased publication of medical-criminological studies on political criminals, revolutionaries, and also anarchists. One of the main objectives of most of these studies was to offer a medical explanation that demonstrated the etiological factors of political crimes. The emergence of many new theories to explain such “revolted individuals” coincided both with the period of radical anarchism seen in the late nineteenth century and an important stage in mental medicine in some European countries, which was instrumental in significantly developing the field of criminology.

## NOTES

1 The group of comrades included Émile Henry (1872-1894), an anarchist militant responsible for having thrown an explosive device into Cafe Terminus, in Paris, in February 1894, for which he was sentenced to death in May of the same year. Like the attack on President Sadi Carnot, the case was reported in the international press, sparking concerns among the governments of several countries (Maitron, 1981).

2 This and the other citations in foreign languages have been freely translated into English.

3 The physicians who signed the medical/legal report on the autopsy and the causes of Carnot’s death were: Antoine Gailleton (1829-1904), Alexandre Lacassagne, Henry Coutagne (1846-1895), Louis Ollier (1830-1900), Ptonce, Lépine, Rebatel, Michel Gangolphe (1858-1919), and Jean Fabre (Lacassagne, 1894, p.72).

4 According to Robert Darntone (2010, p.220-221), the “history of science could prove to be a strategic field for evaluating the game between social history and the history of ideas.”

5 François Claudius Koënigstein (1859-1892), better known as Ravachol, an emblematic figure for the anarchist movement around the world, was responsible for two bombings that shook the Parisian peace. He “was born in the French commune of Saint-Chamond in October 1859. With working-class parents, he had a very humble and troubled childhood, being left in the care of a wetnurse until he was 3. He was then transferred to an institution, where he stayed until the age of 7. As an adult, he lived in several regions of France, wandering from town to town, earning a living however he could, turning his hand to whatever came to hand, which lent him experience in a variety of trades” (Benevides, 2017, p.216-217).

6 For advocates of this legal current, criminal sentences symbolized a kind of retribution for the damage caused to victims of crime. Based on the theory of civil law contracts and the idea of free will as the main basis for punishability, they argued that society was organized on interpersonal relationships of a contractual nature. Therefore, in the event of any non-observance of said “social contract” – namely, committing an offense – the offender would be published by criminal sanctions and thereby make amends for the harm caused to the victim. The penalty would also serve as a deterrent against future lawbreaking (Nye, 1984, p.34; Renneville, 2006, p.32; Fonteles Neto, 2016, p.547; Paula, 2011, p.75-76).

7 According to Mucchielli, the physicians from the last few decades of the nineteenth century reasoned very similarly in Italy, Germany, and England; therefore, it was “hard to imagine why the situation should have been different in France.” According to the author, “the alleged French exception – especially the ‘sociological conception’ ... advanced by some French historians – has already been challenged” (Mucchielli, 2006, p.208; emphasis in the original).

8 Gabriel Tarde and René Garraud made important observations about crime and also anarchy. In relation to Tarde, it is worth mentioning *La criminalité comparée* (1886), in which he highlights the importance of “suggestibility” in the sociological explanations of crime. As for Garraud, his book *L’anarchie et la répression*, published in 1895, is of particular note.

9 A word that means aversion to or mistrust of change, or even hostility towards what is new.

## References

[B1] ALTENA Bert (2016). Anarchism as a social movement, 1870-1940. Sozial.Geschichte Online.

[B2] ALVAREZ Marcos César (2002). A criminologia no Brasil ou como tratar desigualmente os
desiguais. Dados.

[B3] ANSOLABEHERE Pablo (2005). El hombre anarquista delincuente. Revista Iberoamericana.

[B4] ARTIERES Philippe, Mucchielli Laurent (1994). Histoire de la criminologie francaise.

[B5] (1894). ASSASSINAT du président de la République. Le Petit Journal.

[B6] AVELINO Nildo (2010). “Le criminel fin-de-siècle”: psiquiatrização da anarquia no
século XIX. Aurora.

[B7] BANTMAN Constance, Knepper Paul, Johansen Anja (2016). The Oxford handbook of the history of crime and criminal
justice.

[B8] BECKER Peter, Becker Peter, Wetzell Richard F (2006). Criminals and their scientists: the history of criminology in
international perspective.

[B9] BECKER Peter, WETZELL Richard F (2006). Criminals and their scientists: the history of criminology in
international perspective.

[B10] BENEVIDES Bruno Corrêa de Sá e (2017). Feiura como indício de delinquência: uma análise de Ravachol
segundo Cesare Lombroso. Temporalidades: Revista de História.

[B11] CAHM Caroline (1989). Kropotkin and the rise of revolutionary anarchism, 1872-1886.

[B12] CALAFATO Trevor, Knepper Paul, Ystehede Per (2013). The Cesare Lombroso handbook.

[B13] (1894). CARNOT killed. The New York Times.

[B14] COLOMBO Eduardo, Colombo Eduardo, Colson Daniel (2004). História do movimento operário revolucionário.

[B15] COLSON Daniel (2017). Propaganda and the deed: anarchism, violence and the
representational impulse. American Studies.

[B16] DARNTON Robert, Darnton Robert (2010). O beijo de Lamourette: mídia, cultura e revolução.

[B17] DEL OLMO Rosa (2017). A América Latina e sua criminologia.

[B18] DIAS Allister Andrew Teixeira (2015). Arquivos de ciências, crimes e loucuras: Heitor Carrilho e o debate
criminológico do Rio de Janeiro entre as décadas de 1920 e 1940.

[B19] FARRÉ Juan Avilés (2012). Un punto de inflexión en la historia del anarquismo: el congreso
revolucionario de Londres de 1881. Cuadernos de Historia Contemporánea.

[B20] FERLA Luis Antônio Coelho (2005). Feios, sujos e malvados sob medida: do crime ao trabalho, a utopia
médica do biodeterminismo em São Paulo (1920-1945).

[B21] FONTELES Francisco Linhares (2016). A criminologia e a polícia no Brasil na transição do século XIX
para o XX. Passagens: Revista Internacional de História Política e Cultura
Jurídica.

[B22] GIBSON Mary S, Becker Peter, Wetzell Richard F (2006). Criminals and their scientists: the history of criminology in
international perspective.

[B23] GINZBURG Carlo (1989). Mitos, emblemas, sinais: morfologia e história.

[B24] HENZE Martina (2009). Crime on the agenda: transnational organizations
1870-1955. Historisk Tidsskrift.

[B25] HIRSCH Steven, VAN DER WALT Lucien, Hirsch Steven, Van der Walt Lucien (2010). Anarchism and syndicalism in the colonial and postcolonial world,
1870-1940: the praxis of national liberation, internationalism, and social
revolution.

[B26] HOBSBAWM Eric (2012). A era do capital: 1848-1875.

[B27] HORN David G (2003). The criminal body: Lombroso and the anatomy of deviance.

[B28] JACOB Frank, KESSLER Mario, Jacob Frank, Kessler Mario (2021). Transatlantic radicalism: socialist and anarchist exchanges in the 19th
and 20th centuries.

[B29] JENSEN Richard Bach (2004). Daggers, rifles and dynamite: anarchist terrorism in nineteenth
century Europe. Terrorism and Political Violence.

[B30] JENSEN Richard Bach (2001). Criminal anthropology and anarchist terrorism in Spain and
Italy. Mediterranean Historical Review.

[B31] JOLL James (1977). Anarquistas e anarquismo.

[B32] KALIFA Dominique (2019). A tinta e o sangue: narrativas sobre crimes e sociedade na Belle
Époque.

[B33] KALUSZYNSKI Martine, Becker Peter, Wetzell Richard F (2006). Criminals and their scientists: the history of criminology in
international perspective.

[B34] KNEPPER Paul, Triplett Ruth (2017). The Wiley handbook of the history and philosophy of criminology.

[B35] (1894). L’EXECUTION de Caserio. Le Petit Journal.

[B36] LACASSAGNE Alexandre (1894). L’assassinat du président Carnot.

[B37] LANTERI-LAURA Georges, Mucchielli Laurent (1994). Histoire de la criminologie française.

[B38] LOMBROSO Cesare, LASCHI Rodolfo (1890). Il delitto politico e le rivoluzioni in rapporto al diritto,
all’antropologia criminale ed alla scienza di governo.

[B39] LOPREATO Christina Roquette (2003). O espírito das leis: anarquismo e repressão política no
Brasil. Verve.

[B40] MAITRON Jean (1981). Ravachol e os anarquistas.

[B41] MARINI Gualtiero (2017). Revolução, anarquia e comunismo: às origens do socialismo
internacionalista italiano (1871-1876).

[B42] MERRIMAN John (2009). The Dynamite Club: how a bombing in fin-de-siècle Paris ignited the age
of modern terror.

[B43] MONTEIRO Fabrício Pinto (2010). O niilismo social: anarquistas e terroristas no século XIX.

[B44] MUCCHIELLI Laurent, Becker Peter, Wetzell Richard F (2006). Criminals and their scientists: the history of criminology in
international perspective.

[B45] MUSUMECI Emilia (2018). Against the rising tide of crime: Cesare Lombroso and control of
the “dangerous classes” in Italy, 1861-1940. Crime, Histoire et Sociétés.

[B46] NYE Robert A (1984). Crime, madness, and politics in modern France: the medical concept of
national decline.

[B47] PAULA Richard Negreiros de (2011). Paciente duplicado: psiquiatria e Justiça no Rio de Janeiro, entre as
décadas de 1890 e 1910.

[B48] PICK Daniel (1989). Faces of degeneration: a European disorder, c.1848-1918.

[B49] RAFTER Nicole Hahn, POSICK Chad, ROCQUE Michael (2016). The criminal brain: understanding biological theories of crime.

[B50] RÉGIS Emmanuel (1890). Les régicides dans l’histoire et dans le présent: étude
médicopsychologique.

[B51] RENNEVILLE Marc, Becker Peter, Wetzell Richard F (2006). Criminals and their scientists: the history of criminology in
international perspective.

[B52] RENNEVILLE Marc (2005). La criminologie perdue d’Alexandre Lacassagne
(1843-1924). Criminocorpus: Revue d’Histoire de la justice, des crimes et des
peines.

[B53] RENNEVILLE Marc (2003). Crime et folie: deux siècles d’enquêtes médicales et
judiciaires.

[B54] ROMANI Carlo, BENEVIDES Bruno Corrêa de Sá e, Jacob Frank, Kessler Mario (2021). Transatlantic radicalism: socialist and anarchist exchanges in the 19th
and 20th centuries.

[B55] SADI Carnot (1894). O Paiz.

[B56] SALVATORE Ricardo, Triplett Ruth (2017). The Wiley handbook of the history and philosophy of criminology.

[B57] SAMIS Alexandre (2002). Clevelândia: anarquismo, sindicalismo e repressão política no
Brasil.

[B58] SIERRA Álvaro Girón, Miranda Marisa, Sierra Álvaro Girón (2009). Cuerpo, biopolítica y control social: América Latina y Europa en los
siglos XIX y XX.

[B59] SIERRA Álvaro Girón (2003). Kropotkin between Lamarck and Darwin: the impossible
synthesis. Asclepio.

[B60] SPIERENBURG Pieter, Knepper Paul, Johansen Anja (2016). The Oxford handbook of the history of crime and criminal
justice.

[B61] TAIBO Carlos (2018). Anarquistas de ultramar: anarquismo, indigenismo,
descolonización.

[B62] VILLA Renzo, Knepper Paul, Ystehede Per (2013). The Cesare Lombroso handbook.

[B63] WETZELL Richard F (2000). Inventing the criminal: a history of German criminology,
1880-1945.

[B64] WOODCOCK George (2006). História das ideias e movimentos anarquistas: o movimento.

